# Bioinspired ZnS:Gd Nanoparticles Synthesized from an Endophytic Fungi *Aspergillus flavus* for Fluorescence-Based Metal Detection

**DOI:** 10.3390/biomimetics4010011

**Published:** 2019-02-01

**Authors:** Priyanka Uddandarao, Raj Mohan Balakrishnan, Apoorva Ashok, Sai Swarup, Priti Sinha

**Affiliations:** Department of Chemical Engineering, National Institute of Technology Karnataka, Karnataka 575025, India; uddandaraopriyanka@gmail.com (P.U.); apoorvaashok5@gmail.com (A.A.); ssbharti1996@gmail.com (S.S.); pritisinha106@gmail.com (P.S.)

**Keywords:** *Aspergillus flavus*, biosynthesis, endophytic fungi, semiconductor, ZnS nanoparticles

## Abstract

Recently, several nonconventional sources have emerged as strong hotspots for the biosynthesis of chalcogenide quantum dots. However, studies that have ascertained the biomimetic methodologies that initiate biosynthesis are rather limited. The present investigation portrays a few perspectives of rare-earth(Gd)-doped ZnS biosynthesis using the endophytic fungi *Aspergillus flavus* for sensing metals based on their fluorescence. Analysis of ZnS:Gd nanoparticles was performed by elemental analysis, energy-dispersive X-ray spectroscopy (EDS), atomic force microscopy (AFM), X-ray diffraction (XRD), Fourier-transform infrared spectroscopy (FTIR), photoluminescence (PL), and transmission electron microscopy (TEM). The results of TEM demonstrated that the particles were polycrystalline in nature, with a mean size of 10–18 nm. The fluorescence amenability of the biogenic ZnS nanoparticles was further used for the development of a simple and efficient sensing array. The results showed sensitive and detectable quenching/enhancement in the fluorescence of biogenic colloidal ZnS nanoparticles, in the presence of Pb (II), Cd (II), Hg (II), Cu (II) and Ni (II), respectively. The fluorescence intensity of the biogenic ZnS:Gd nanoparticles was found to increase compared to that of the ZnS nanoparticles that capacitate these systems as a reliable fluorescence sensing platform with selective environmental applications.

## 1. Introduction

Zinc sulphide (ZnS) is used in optical sensitizers, optical sensors, ultraviolet (UV) light sensors, chemical sensors, biosensors, nanogenerators, electroluminescent materials, field emitters, and field effect transistors [1,2]. Biological nanoparticles are encapsulated with protein capping, which helps them in providing discrete nanoparticles. The biological entities used for the synthesis secrete proteins that impart surface modification, thereby providing good stability and improved characteristics. The nanoparticle yield is higher in fungi compared to plants and bacteria, as they secrete large amounts of proteins, which directly influence productivity. They exhibit monodispersity with well-defined dimensions using minimal media requirements. In addition, their production can be easily scaled up from laboratory to industrial scale [3]. Endophytes are microbes residing in the internal parts of plant tissues. They are known to secrete a rich source of metabolites and are reported to perform multiple activities in the field of medicine, with agriculture and industry potentials. 

The use of endophytes for the synthesis of nanomaterials has been reported in the literature [4]. The integration of nanomaterials from biological origin in nanoengineered devices has also gained significant research interest. The biogenic synthesis of quantum dots is usually preferred over the traditional chemical methods due to their nontoxic nature and inherent biocompatibility to the microbial environment [5]. Chemically synthesized nanoparticles are highly unstable and tend to agglomerate in the absence of suitable trapping media, whereas biological synthesis provides discrete nanoparticles through surface modification with protein capping [6]. This imparts long-term stability and improves their optical and electronic properties [7]. The yield of the nanoparticles produced by fungi is reported to be high compared to that of plants and bacteria, due to the fact that fungi secrete a larger amount of proteins which directly influence the productivity of the nanoparticles. Furthermore, good monodispersity, minimal media requirements, intracellular metal uptake capabilities, well-defined dimensions, and ease of scaling up the productivity are some of the advantages of nanoparticles synthesized from fungi [3].

The discharge of heavy metals from various industrial activities to the water bodies poses a severe threat to the environment. In addition, heavy metals have been shown to cause detrimental health effects on all life forms because the metals easily bioaccumulate and biomagnify in the food chain [8,9,10]. Conventional analytical techniques used to quantify these metals include atomic fluorescence spectroscopy (AFS), atomic absorption spectroscopy (AAS), inductively coupled plasma optical emission spectrometry (ICP-OES), inductively coupled plasma mass spectrometry (ICP-MS), and high-performance liquid chromatography (HPLC). Although these techniques can precisely determine the concentration of metals, there is still an urgent need to have techniques that can instantly quantify the metal concentrations in actual field conditions, e.g., in water bodies. In this line of progressive analytical research, the synergy between biotechnology and nanotechnology has led to innovation in developing biosensors that are species-selective and efficient under harsh environmental conditions [11,12,13]. Semiconductor nanocrystals have exhibited extraordinary properties, and they are applied in many emerging research areas. Chalcogen-based semiconductor nanoparticles, generally called quantum dots, are known as predominant components for fluorescence-based materials and bioimaging due to their optical properties that account for their size tunable quantum confinement effects. Their manufacture and use at the nanoscale has gained interest from the industrial and academic community [14,15].

Despite the progress in the biosynthesis of chalcogenide quantum dots, the conceivable outcomes of these nanofabrications, particularly ZnS, have not been studied so far. In recent years, surface passivation of nanoparticles has interested researchers, which helps in the progress of nanophosphors of required sizes, where the surface gets passivated and enhances the properties [16,17]. Zinc sulfide doping with uncommon rare-earth metals is considered an attractive strategy for the improvement of photoluminescence (PL) properties, because such uncommon rare-earth metals have a unique electronic structure, and this characteristic is important for the production of charge exchangers [18,19,20,21]. The present study focuses on the biosynthesis of Gd-doped ZnS and the fluorescence detection of metals. A simple, sensitive, and inexpensive method was developed to detect and remove heavy metals using ZnS:Gd nanoparticles.

## 2. Materials and Methods

### 2.1. Microbial Synthesis of Gd-Doped ZnS Nanoparticles

In the present study, the endophytic fungi *Aspergillus flavus* was isolated from leaf segments of *Nothapodytes foetida* collected from the Agumbe forest located at 13°30″ N and 75°2″ E in the Western Ghats, Daksina Karnataka, India. Briefly, the isolated cultures were maintained on potato dextrose agar (PDA) medium, at room temperature, and were subcultured at monthly time intervals. The mycelial discs (2 mm × 5 mm) from an actively growing source culture of *A. flavus* were aseptically transferred into 250 mL potato dextrose broth (PDB) and grown for two days at 28 °C and 115 rpm (Troemner, Thermo Fisher Scientific, Ottawa, ON, Canada). Thereafter, the media components were drained off and the fungal biomass was harvested and added to a flask containing 100 mL of 3 mM zinc sulfate heptahydrate solution in order to facilitate the biosynthesis of ZnS nanoparticles. Synthesis of ZnS:Gd nanoparticle was carried out by modifying a previous protocol [22,23] by adding 0.3 M gadolinium nitrate, stirred for 10 min at room temperature along with the zinc precursor. After the synthesis, the samples were characterized using energy-dispersive X-ray spectroscopy (EDS), elemental mapping (JEM-2100, JEOL, Tokyo, Japan), and atomic force microscopy (AFM) (Alpha300RA AFM, WITec GmbH, Ulm, Germany) for the comparison of ZnS and ZnS:Gd nanoparticles. Furthermore, the optical and structural properties of ZnS:Gd nanoparticles were analyzed using fluorescence spectroscopy (Cary Varian Eclipse fluorescence spectrophotometer, Agilent Technologies, Braeside, VIC, Australia), Fourier-transform infrared spectroscopy (FTIR) analysis (JASCO spectrometer 4100, Perkin–Elmer, Waltham, MA, USA), X-ray diffraction analysis (XRD) (JEOL, DX GE-2P vertical goniometer), and a transmission electron microscope (TEM) (JEM 2100, JEOL, Pleasanton, CA, USA).

### 2.2. Detection of Metals by ZnS and ZnS:Gd Nanoparticles

Standard stock solutions of 1 M Cd (II), Cu (II), Ni (II), Hg (II), and Pb (II) were prepared by dissolving an appropriate amount of the respective precursor salts with water in a volumetric flask. The concentration of the metal tested was 100 µM. One milliliter of each metal ions solution was added to 9 mL of ZnS nanoparticles (2.6 mM concentration) solution and stirred for 20 min, and thereafter transferred to a fluorescent cuvette. The fluorescent intensity of the solution was recorded from 300 to 650 nm, with the excitation wavelength fixed at 315 nm using fluorescence spectroscopy (Fluorolog 3 TCSPC, Horiba, Japan) [24,25,26].

## 3. Results and Discussion

### 3.1. Comparison of ZnS and ZnS:Gd Nanoparticles Based on EDS, Elemental Analysis, and AFM

**Figure 1:**
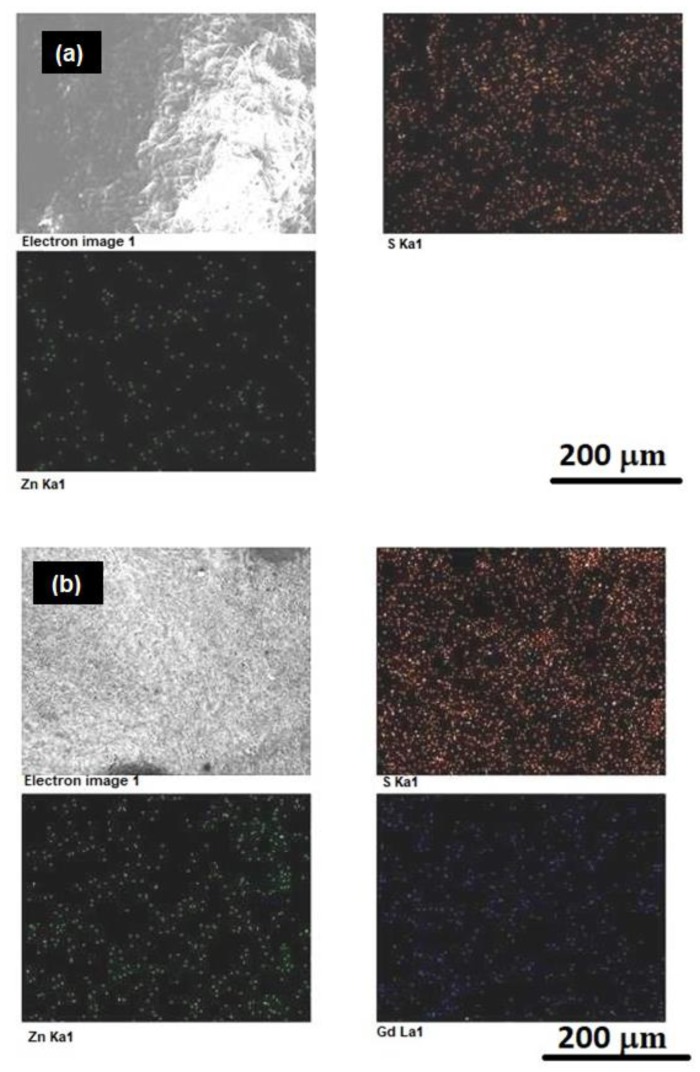
Elemental mapping of (**a**) ZnS and (**b**) ZnS:Gd (3%) nanoparticles.

**Figure 2:**
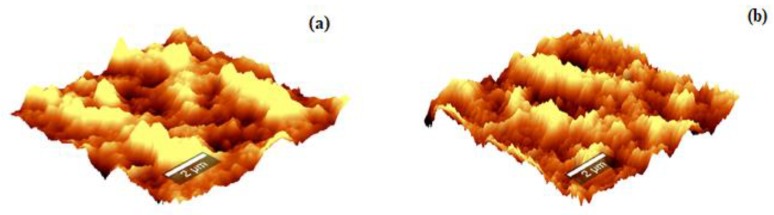
AFM analysis of (**a**) ZnS, and (**b**) ZnS:Gd nanoparticles.

Supplementary Figure S1a,b shows the EDS spectra of ZnS and ZnS:Gd biogenic nanoparticles. The presence of gadolinium in the doped samples was examined by EDS of ZnS:Gd (3%) nanoparticles, showing only gadolinium, zinc, and sulfur signals. Furthermore, detailed analysis of the chemical composition of the ZnS (Figure 1a) and ZnS:Gd (Figure 1b) was performed by elemental mapping. The presence of C, O, Zn, S, and Gd was observed. The graph indicates that Zn, S, and Gd was uniformly distributed; however, S was widely distributed. The images show that Gd doped on the surface of the ZnS:Gd (3%) nanoparticles as the optimum concentration [27,28]. The morphology properties of the nanostructures were studied by AFM, as shown in Figure 2a,b. As seen from these AFM images, the distribution of the nanoparticles on the substrate changed with Gd doping. This distribution caused a change in the surface roughness from 53.8 to 88.2 nm with the addition of Gd [29]. This change indicates that the Gd atoms were replaced into the ZnS sites.

### 3.2. Morphological and Optical Characterization

**Figure 3:**
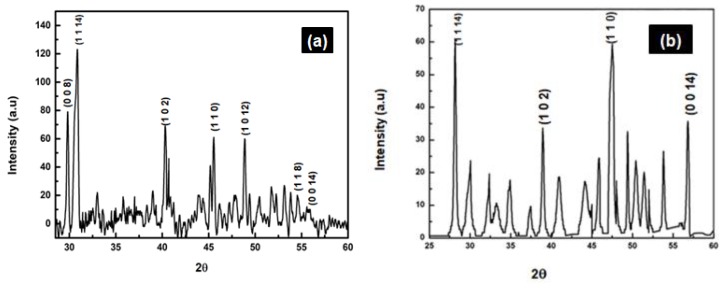
XRD of (**a**) ZnS:Gd (3%) and (**b**) ZnS nanoparticles. Reprinted from [22], with permission from Elsevier. a.u.: Arbitrary units.

In order to determine the phase of doped nanoparticles, we performed XRD analysis of ZnS and ZnS:Gd nanoparticles. Figure 3a shows the XRD pattern of ZnS:Gd nanoparticles prepared with 3% Gd. The nanocrystalline nature of the samples resulted in the broadening of XRD peaks. The incorporation of Gd ions as substituents in ZnS host lattice resulted in a small shift in the XRD peaks in the spectra towards a lower diffraction angle with Gd doping concentration (3%) compared with XRD of ZnS nanoparticles (Figure 3b) [22]. It was reported that the dopant atoms in the ZnS host lattice caused lattice disorder, which was seen when the intensity of the prominent peak changed with Gd doping compared with a normal ZnS nanoparticle pattern. The broadening of XRD peaks of the prepared samples indicated the nanocrystalline nature of the samples. The spectra showed a small shift/additional peaks in the XRD (0 0 8), (1 1 14), (1 0 2), (1 1 0), (1 0 12), (1 1 8), and (0 0 14) towards a lower diffraction angle with Gd doping concentration, suggesting the incorporation of Gd ions as substituents in the ZnS host lattice compared with the undoped XRD (i.e., peaks at (1 1 14), (1 0 2), (1 1 0), and (0 0 14)). Moreover, the change in the intensity of the prominent peak with Gd concentration, which could be due to the lattice disorder, was caused by the presence of dopant atoms in the ZnS host lattice. The XRD data of ZnS and ZnS:Gd nanoparticles show diffraction peaks centered at 27.85°, 28.45°, 39.61°, 47.54°, 52.08°, 57°, and 28.45°, 39.61°, 47.54°, and 57° (2θ) which can be well-indexed to the hexagonal phase of ZnS (Joint Committee on Powder Diffraction Standards JCPDS card No. 01-089-2195) [23].

**Figure 4:**
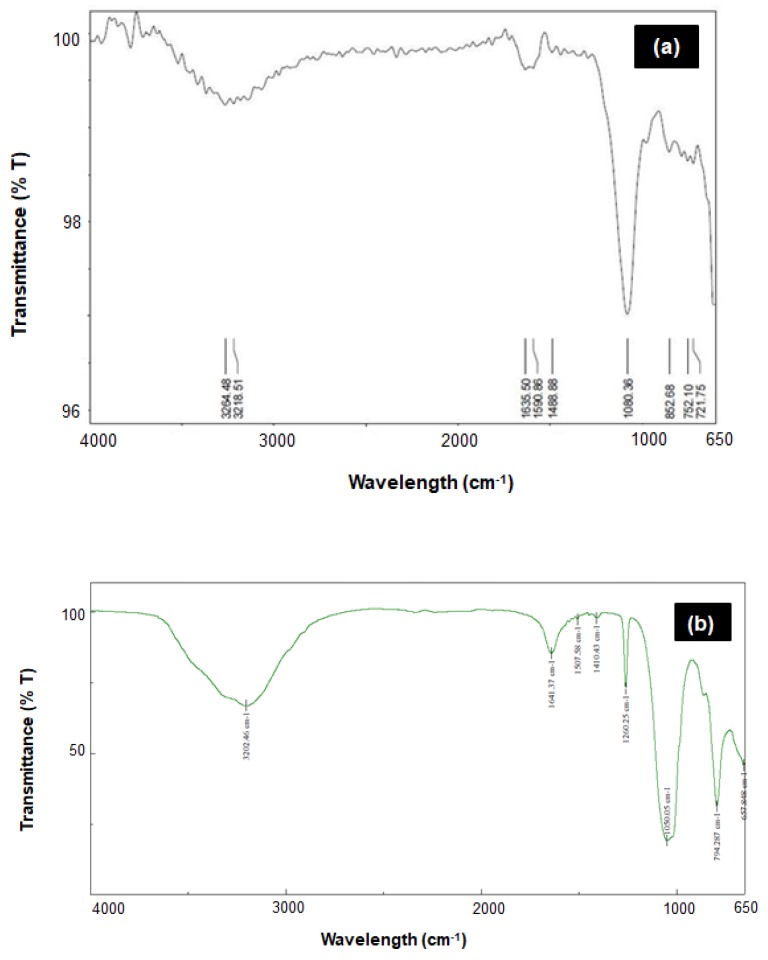
FTIR spectra of (**a**) ZnS:Gd (3%) and (**b**) ZnS nanoparticles. Reprinted from [22], with permission from Elsevier.

**Figure 5:**
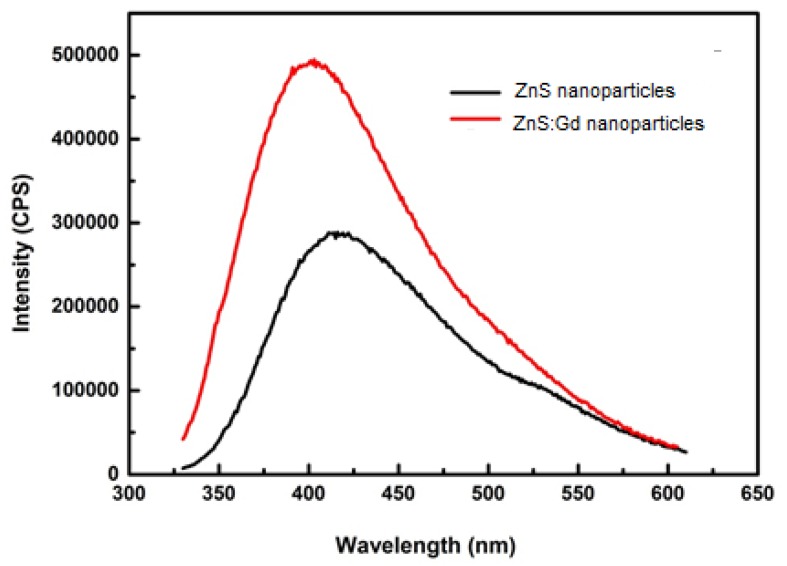
Photoluminescence spectra of ZnS and ZnS:Gd (3%) nanoparticles. CPS: Counts per second. Reprinted from [30], with permission from Elsevier.

Based on the FTIR spectrum of ZnS:Gd nanoparticles (Figure 4a), the spectral peaks 721, 752, and 852 cm^−1^ correspond to C–H bending. Furthermore, the peaks at 1080 and 1488 cm^−1^ correspond to C–O stretching and C–H bending, respectively; whereas the peaks at 1590 and 1635 cm^−1^ correspond to C–C double bond stretching, and the peaks at 3218 and 3264 cm^−1^ correspond to C–H stretching. An increase in the number of peaks and peak shift was observed for the ZnS:Gd nanoparticles compared to ZnS nanoparticles (Figure 4b) [22]. The nanoparticles synthesized by this method consist of proteins capping the nanoparticle surface via the thiol group of cysteine and methionine residues. The presence of C–H, C–O, and C–C bonds was due to the amine groups of proteins present on the outer surface of the nanoparticles confirming the presence of sulfur-bearing bonds. Figure 5 shows the PL intensity of ZnS:Gd nanoparticles, recorded with an excitation wavelength of 315 nm. Both the ZnS nanoparticle and Zns:Gd nanoparticle have emission peaks at 410 nm; however, the intensity of the Gd-doped ZnS nanoparticle was more than that of the ZnS nanoparticles. The ZnS:Gd (3%) showed the highest fluorescence efficiency compared to the nondoped samples (Figure 5) [30]. The substitution of Zn^2+^ ions with Gd^3+^ ions might increase the defect sites and produce new radiation centers, which enhances the fluorescence efficiency. Fluorescence enhancement may be induced by the higher density of interstitial Zn trap states as well as nonradiative centers at 3% dopant concentration. Therefore, these samples have potential applications in optoelectronic devices [31,32], such as optical detectors, solar cell field-effect transistors, photonics, luminescence sensors, light-emitting devices (LEDs), low-threshold lasers, optical amplifiers, and biological probes.

**Figure 6:**
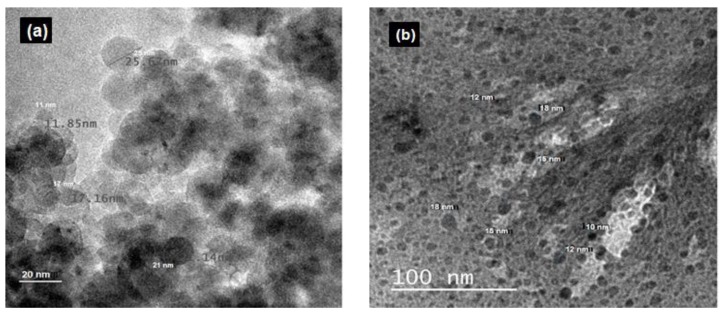
TEM micrographs of (**a**) ZnS (reprinted from [22], with permission from Elsevier) and (**b**) ZnS:Gd (3%) nanoparticles.

The morphology and particle diameter of the synthesized ZnS and ZnS:Gd (0 and 3%) semiconductor particles were examined using TEM. The results revealed the formation of monodispersed nanoparticles with a spherical shape and a narrow size distribution. The estimated average mean diameter of the synthesized ZnS ranged from 12–24 nm (Figure 6a) and ZnS:Gd (3%) nanoparticles were 10–18 nm in diameter (Figure 6b) [22,23,33]. 

### 3.3. Sensing of Metals Using ZnS Nanoparticles

**Figure 7:**
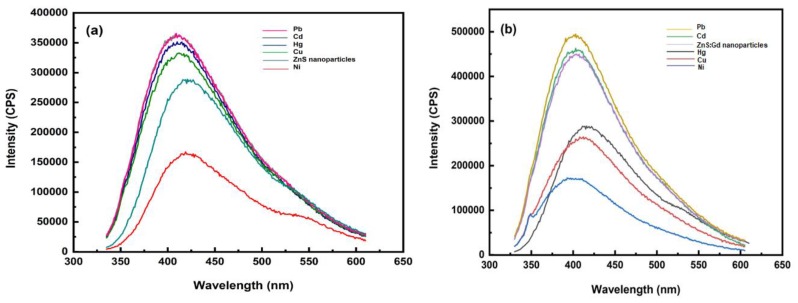
Photoluminescence spectra of heavy metal solution with (**a**) ZnS nanoparticles and (**b**) ZnS:Gd nanoparticles. CPS: counts per second.

The interaction of ZnS nanoparticles with heavy metals like Pb (II), Cd (II), Hg (II), Cu (II), and Ni (II) absorbs at 315 nm, which has been used as the excitation wavelength for PL studies [34]. It has been shown that the photoluminescence of ZnS nanoparticles is usually accomplished by the coordination of metal ions to ZnS nanoparticles forming a complex [35]. Thus, it is inferred that the long-range interaction of ZnS nanoparticles around the metal ions relieves the excitonic barrier, which is responsible for the photoluminescence quenching in some metals and is well used in fast qualitative sensing of metal ions present in the aqueous medium [36]. Photoluminescence results for ZnS nanoparticles was found to give maximum intensity for Pb (II) and Cd (II), whereas the minimum intensity was recorded for Ni (II) (Figure 7a). Furthermore, the intensity was enhanced in the case of Pb (II), Cd (II), Hg (II), and Cu (II) ions. Gadolinium-doped ZnS nanoparticles showed enhancement of the photoluminescence spectra for Pb (II) and Cd (II), and quenched for Hg (II), Cu (II), and Ni (II) ions (Figure 7b) [37,38,39]. It has been previously reported that Cu^2+^ ions quenched the fluorescence intensity of the graphene quantum dots by a magnitude of 2–3 times in comparison to other tested metal ions like Fe, Al, Co, Cd, and Pb [40]. The substitution of Zn^2+^ ions with Gd^3+^ ions might augment the defect sites and produce new radiation centers, which enhances the fluorescence efficiency [41].

## 4. Conclusions

In the present study, the doping effect of Gd on ZnS nanoparticles was studied. Gadolinium was used as a dopant because of its half-filled electron configurations. Furthermore, the Gd component has a half-filled electronic configuration 4f-shell containing seven electrons and a void electronic configuration 5d-shell, which are unique when compared to other uncommon rare-earth metals. If a metal is used as a dopant, then only the lattice parameters change and peak shifts, an increase in intensity depending on the doping level, and differences in ionic radii can be observed. Moreover, the presence of Gd was confirmed by EDS and elemental mapping. Research on environmental sensors has gained widespread interest because of their application in manufacturing novel nanosized sensors for pollutant detection. Future perspectives can be the improvement in the sensitivity of the probe by surface functionalization, i.e., by coating nanoparticles onto a polymer or by encapsulating within a silica shell and immobilizing on the tip of an optical fiber by a polymer coating.

The present study reports the biosynthesis of Gd-doped ZnS nanoparticles using an endophytic fungus *A. flavus*. The results showed that the synthesized ZnS:Gd nanoparticles exhibited a hexagonal phase and, according to TEM analysis, the size distribution was in the range of 10–18 nm. A significant increase in the intensity of XRD and PL was observed in ZnS:Gd nanoparticles when compared to nondoped ZnS nanoparticles. Furthermore, the biosensing capacity of ZnS nanoparticles was found to enhance with Gd dopant. The biological nanocatalysts synthesized in this study offer a novel catalytic and biological model in the field of material science.

## Supplementary Materials

The following are available online at https://www.mdpi.com/2313-7673/4/1/11/s1, Figure S1: EDS spectra of (a) ZnS nanoparticles and (b) ZnS nanoparticles with 3% Gd dopant concentration.
