# Supplementary material for: Bioinspired ZnS:Gd Nanoparticles Synthesized from an Endophytic Fungi Aspergillus flavus for Fluorescence-Based Metal Detection
Source: Biomimetics (Basel). 2019 Feb 1;4(1):11. doi: 10.3390/biomimetics4010011 (PMC6477624; doi:10.3390/biomimetics4010011)
Supplement: Supplementary file 1 [file biomimetics-04-00011-s001.pdf]

Biomimetics **2019**, *4*, 11

Supplementary Materials: Bioinspired ZnS:Gd Nanoparticles Synthesized from an Endophytic Fungi *Aspergillus flavus* for Fluorescence-Based Metal Detection

Priyanka Uddandarao, Raj Mohan Balakrishnan *, Apoorva Ashok, Sai Swarup and Priti Sinha

Department of Chemical Engineering, National Institute of Technology Karnataka, Karnataka 575025, India; uddandaraopriyanka@gmail.com (P.U.); apoorvaashok5@gmail.com (A.A.); ssbharti1996@gmail.com (S.S.); pritisinha106@gmail.com (P.S.)

* Correspondence: rajmohanbala@gmail.com; Tel.: +91-973-993-9986

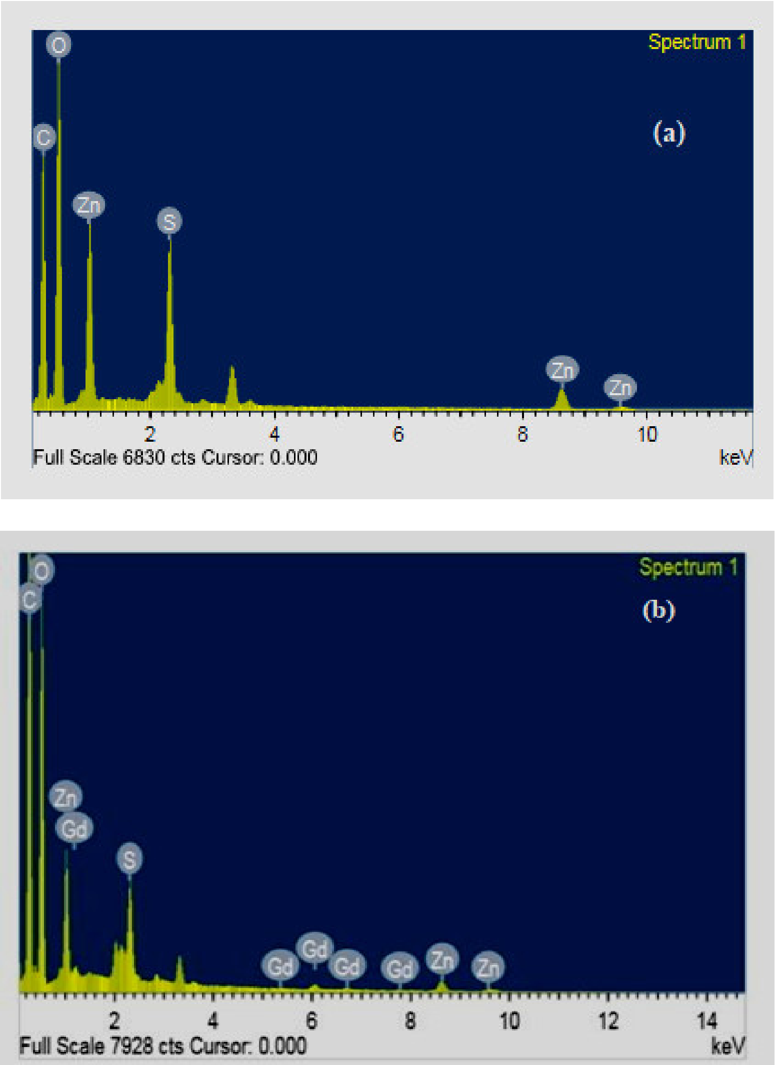

Figure S1. EDS spectra of (**a**) ZnS nanoparticles and (**b**) ZnS nanoparticles with 3% Gd dopant concentration.
